# Telehealth-Based Cardiac Rehabilitation for Heart Failure: A Systematic Review of Effectiveness, Access, and Patient-Centred Outcome

**DOI:** 10.3390/medicina62010025

**Published:** 2025-12-23

**Authors:** Abdulfattah S. Alqahtani

**Affiliations:** Department of Rehabilitation Health Sciences, College of Applied Medical Sciences, King Saud University, Riyadh 12372, Saudi Arabia; abalqahtani@ksu.edu.sa; Tel.: +966-114678534

**Keywords:** heart failure, telehealth, cardiac rehabilitation, exercise capacity, quality of life, adherence

## Abstract

*Background and Objectives*: Heart failure (HF) affects millions globally, with traditional cardiac rehabilitation (CR) improving outcomes but facing access barriers. Telehealth-based CR offers a promising alternative, yet its effectiveness and patient-centred outcomes require updated evaluation. This systematic review aimed to assess the effectiveness, accessibility, and patient-centred outcomes of telehealth-based CR compared with usual care or centre-based CR in adults with HF. *Materials and Methods*: This systematic review followed PRISMA 2020 guidelines. Eligible studies were randomized controlled trials involving adults with HF receiving telehealth CR (e.g., telephone, apps, remote monitoring) compared with usual care or centre-based CR; non-RCTs and studies lacking relevant outcomes were excluded. Searches of PubMed, Medline, CINAHL, EMBASE, and Web of Science identified studies published between 2020–2025. Primary outcomes were exercise capacity (six-minute walk distance [6MWD], peak VO_2_) and quality of life (QoL); secondary outcomes included adherence, satisfaction, and clinical events. Meta-analyses used standardized mean differences (SMD) for 6MWD and QoL. Risk of bias was assessed using PEDro, Jadad, and RoB2 tools. *Results*: Fourteen randomized controlled trials (total *n* = 7371 participants) met the inclusion criteria. Telehealth CR significantly improved 6MWD (SMD 0.35, 95% CI 0.15–0.55, *p* < 0.001; 6 studies) and QoL (SMD 0.28, 95% CI 0.10–0.46, *p* = 0.002; 8 studies) compared to usual care, showing equivalence to center-based CR. Adherence ranged from 70–92% and satisfaction 75–96%, and hospitalizations declined in some studies, though mortality benefits were not observed. *Conclusions*: Telehealth CR is effective, accessible, and patient-centred for individuals with HF, performing comparably to centre-based CR and better than usual care. It should be integrated into standard HF management, supported by policy and technology investment. Evidence is limited by short follow-up durations and moderate heterogeneity among trials.

## 1. Introduction

Heart failure (HF) is a complex clinical syndrome characterized by impaired cardiac function resulting in inadequate systemic perfusion, exercise intolerance, and reduced quality of life (QoL) [1]. In the United States, HF affects approximately 6.2 million adults, with a higher prevalence among men, African Americans, and older adults. Globally, it impacts more than 26 million individuals, with costs projected to rise by 127% by 2030 [2]. Despite advances in pharmacotherapy and device-based therapies, HF remains a leading cause of hospital readmission, functional decline, and mortality [3,4].

Exercise-based cardiac rehabilitation (CR) is a cornerstone of HF management, improving functional capacity, symptoms, and QoL, and reducing hospital readmissions [5]. However, participation in traditional centre-based CR remains consistently low—often below 30%—due to substantial barriers including transportation difficulties, geographic distance, work commitments, limited programme availability, and caregiver dependency [6,7]. These longstanding access challenges highlight the need for alternative CR delivery models.

Telehealth-based CR (cardiac telerehabilitation) has emerged as a promising solution to these barriers. Modern telehealth CR incorporates telephone consultations, videoconferencing, mobile health applications, wearable sensors, and remote monitoring technologies to deliver supervised exercise training, education, behavioural counselling, and clinical surveillance outside traditional clinical settings [7,8,9,10,11]. The COVID-19 pandemic accelerated global adoption of digital rehabilitation models, supported by rapid advancements in wearable biometrics, remote ECG monitoring, automated feedback systems, and secure digital platforms. These innovations have transformed telerehabilitation from basic telephone follow-up into comprehensive, technology-enabled rehabilitation programmes capable of individualized exercise prescription and continuous oversight [9,10,11,12].

Despite technological progress, substantial heterogeneity persists across telehealth CR interventions with respect to digital platforms, exercise intensity, supervision level, adherence monitoring, and outcome assessment. Earlier systematic reviews were predominantly based on pre-2020 trials, many of which used limited telemonitoring approaches, small sample sizes, and minimal integration of modern digital tools. As a result, existing reviews do not adequately represent the contemporary landscape of app-based, sensor-enhanced, hybrid, or AI-supported telehealth CR models.

Existing systematic reviews do not capture the most recent post-2020 randomized trials or evaluate differences across modern telehealth modalities, leaving uncertainty about the contemporary effectiveness, patient-centred outcomes, and comparative performance of telehealth-based cardiac rehabilitation versus centre-based CR and usual care.

Therefore, there is a pressing need to synthesize the latest randomized controlled trials (RCTs) published between 2020 and 2025 to determine whether contemporary telehealth CR improves exercise capacity, cardiac function, QoL, adherence, and satisfaction relative to usual care and centre-based CR.

This systematic review aimed to evaluate the effectiveness, accessibility, and patient-centred outcomes of telehealth-based cardiac rehabilitation compared with centre-based CR or usual care among adults with heart failure, using RCTs published between 2020 and 2025. Specifically, it examined whether telehealth CR improves exercise capacity, cardiac function, quality of life (QoL), adherence, and satisfaction compared with in-person CR or usual care. We hypothesized that telehealth CR would yield comparable clinical benefits to centre-based CR while providing superior access and adherence.

## 2. Materials and Methods

### 2.1. Study Design

This systematic review and meta-analysis followed the Preferred Reporting Items for Systematic Reviews and Meta-Analyses (PRISMA 2020) guidelines and adhered to Cochrane methodological standards [13,14]. The protocol was prospectively registered in PROSPERO (PROSPERO ID: CRD420251088298, dated: 6 July 2025, Available from https://www.crd.york.ac.uk/PROSPERO/view/CRD420251088298, accessed on 16 August 2025). The primary aim was to synthesize randomized controlled trials (RCTs) evaluating the effectiveness, accessibility, and patient-centred outcomes of telehealth-based cardiac rehabilitation (CR) in adults diagnosed with heart failure.

Telehealth CR was broadly defined as any structured cardiac rehabilitation intervention delivered wholly or predominantly through remote communication technologies, including telephone-supported CR, videoconferencing, mobile health applications, wearable sensor–integrated CR, remote monitoring systems, or hybrid telehealth programs.

### 2.2. Search Strategies

The author, assisted by two trained reviewers, conducted a comprehensive electronic search across four major databases: PubMed/Medline, EMBASE, CINAHL, and Web of Science for studies from 2020–2025. Search terms combined MeSH headings and free-text keywords for heart failure, cardiac rehabilitation, exercise training, and telehealth/telerehabilitation. Boolean operators and truncations were applied.

For PubMed, the following search strategy was used: (“Heart Failure” [Mesh] OR “heart failure” OR “cardiac failure” OR “congestive heart failure”) AND (“Cardiac Rehabilitation” [Mesh] OR “cardiac rehabilitation” OR “exercise training”) AND (telehealth OR telemedicine OR telerehabilitation OR “remote monitoring” OR “mobile health” OR mHealth OR “eHealth”) AND (randomized OR randomised OR “randomized controlled trial” OR RCT). Full database-specific strategies are provided in Supplementary Table S1, ensuring full reproducibility. Duplicates were removed using EndNote X9, and all search records were archived for audit trail.

### 2.3. Selection Criteria (PICOS Framework)

Potential articles were included based on the following criteria: We included studies meeting all of the following criteria (PICOS framework): (1) Population: Adults (≥18 years) with heart failure (any etiology, NYHA class I–IV, acute or chronic phase); (2) Intervention: Telehealth-based cardiac rehabilitation–structured exercise training (aerobic ± resistance) and multidisciplinary HF management delivered predominantly via remote communication (telephone, video, internet/app, or remote monitoring devices). Interventions could be entirely home-based or hybrid (combining initial in-person sessions with follow-up telerehab); (3) Comparison: Either (a) usual care with no structured rehabilitation (standard medical therapy), or (b) a traditional center-based CR program (on-site supervised exercise); (4) Outcomes: At least one of the following reported–exercise capacity (e.g., 6-min walk distance [6MWD], peak VO_2_), health-related quality of life (HRQoL), functional class, program adherence (e.g., session completion rate), patient satisfaction, or clinical events (HF hospitalizations, mortality); (5) Study design: Randomized controlled trials published in English.

Additionally, case reports, editorials, letters to the editor, and conference proceedings were excluded due to the potentially high risk of bias. We also excluded trials in which the intervention was not a true rehabilitation program (e.g., remote monitoring only without exercise or education component), as well as trials focused on other cardiac conditions (e.g., telerehab after myocardial infarction) or mixed-population studies that did not report HF subgroup results.

The author, with assistance from two trained reviewers, screened titles/abstracts and full texts in duplicate. Disagreements were resolved by discussion, with the author acting as adjudicator.

### 2.4. Data Extraction

Data extraction was performed in duplicate using a standardized form to collect information on study design, participant characteristics, intervention details, comparator groups, outcomes, and follow-up durations. The data extracted from each study included authors, year of publication, country, sample size, patient characteristics, intervention, and study duration.

**Figure 1:**
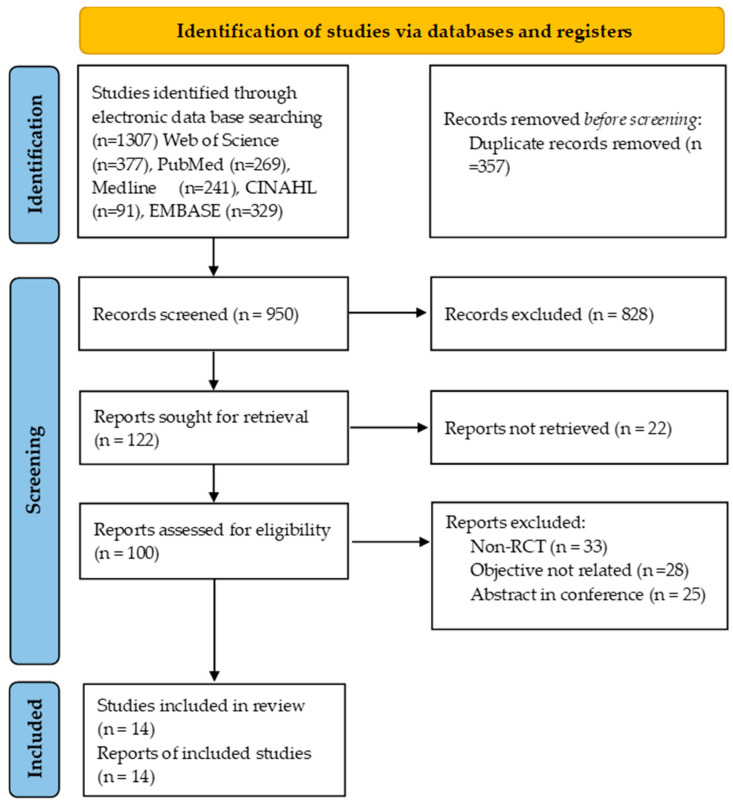
PRISMA flow chart of studies that are eligible for systematic review.

The PRISMA flow chart (Figure 1) illustrates the process of study identification, screening, exclusion, and inclusion.

### 2.5. Quality Assessment

Methodological quality was assessed using two complementary approaches. First, the PEDro scale (0–10) was applied as a summary quality index commonly used in physiotherapy and rehabilitation research, capturing key features such as randomization, allocation concealment, and follow-up [15]. Second, the Cochrane Risk of Bias 2 (RoB 2) tool was used as the primary, domain-based assessment of bias in randomized trials, in line with current Cochrane guidance [16]. Two trained reviewers scored trials independently and reconciled any differences by discussion, with the author acting as adjudicator. The Jadad scale was not used as an independent quality judgment but only to define a threshold for sensitivity analyses, in which we repeated the meta-analyses after excluding trials with Jadad score < 3 [17,18].

### 2.6. Data Synthesis and Analysis

Qualitative synthesis summarized effectiveness (exercise capacity, cardiac function), accessibility (adherence), and patient-centered outcomes (QoL, satisfaction). Meta-analysis was conducted for 6MWD and QoL (SF-36, KCCQ, MLHFQ) using RevMan 5.4, with standardized mean differences (SMD) and 95% confidence intervals (CI). Because higher scores on the MLHFQ indicate worse quality of life, MLHFQ scores were multiplied by−1 before calculating standardized mean differences so that all QoL instruments were aligned in the direction ‘higher scores = better quality of life’ prior to pooling. Heterogeneity was assessed via I^2^. Random-effects models were used due to expected clinical heterogeneity. Subgroup analyses explored telehealth modalities (e.g., app-based vs. telephone). Sensitivity analyses excluded high-risk-of-bias studies (Jadad < 3).

Risk of bias was assessed using the Cochrane RoB 2 tool (Version 2, 2019) for RCTs, implemented via the RoB 2 Excel Macro Form (Beta Version 7). Summary tables and traffic light plots were generated using the Excel tool.

## 3. Results

### 3.1. Study Selection

Figure 1 shows the PRISMA flow chart. Of 1307 records, 14 randomized controlled trials met the inclusion criteria, comprising a total of 7371 patients, with sample sizes ranging from 56 to 1538.

### 3.2. Study Characteristics

Table 1 summarizes the 14 included studies (2020–2025, conducted in Poland, Germany, Denmark, Japan, South Korea, Norway, United States, and international settings). Patients were predominantly male (50–88.6%), with mean ages 62.4–70.3 years, NYHA class I–III, and LVEF ≤ 40–50%. Interventions included hybrid telerehabilitation, remote patient monitoring, mobile apps, and telemonitoring, with durations of 9 weeks to 12 months. Controls were usual care or center-based.

### 3.3. Quality Assessment

**Figure 2:**
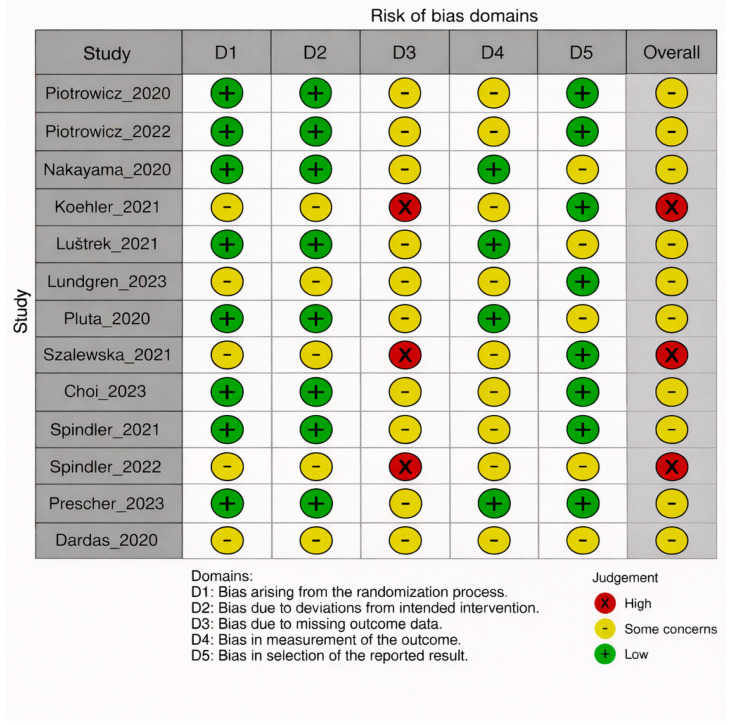
The domain-wise and overall risk of bias assessment for the included studies using the RoB 2 tool [19,20,21,22,23,24,25,26,27,28,29,30,31].

**Figure 3:**
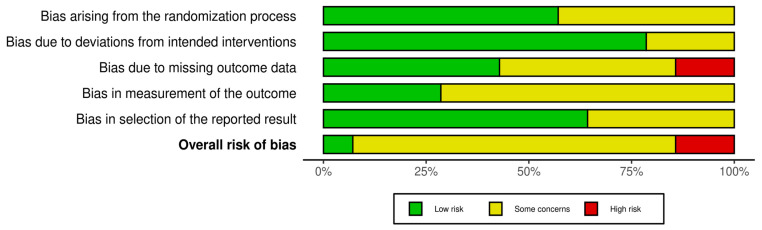
The proportion of studies rated as low risk, some concerns, or high risk across RoB 2 domains and overall bias.

Table 2 summarizes the methodological quality of the included studies based on the PEDro (scores ranging from 6 to 8), Jadad (scores from 1 to 4), and RoB 2 assessments. Overall, the studies were of moderate to high quality, with strengths in randomization procedures and outcome reporting. The nature of exercise-based interventions limited the feasibility of blinding participants and therapists, which contributed to lower Jadad scores, with a mean score of 3.1. According to the RoB 2 evaluation, 11 of the 14 randomized trials were judged at low risk of bias and 3 at some concerns, mainly related to missing outcome data or incomplete reporting. The risk of bias Figure 2 and Figure 3 further illustrate that most studies had a low risk in domains related to randomization and reporting, while some concerns were noted in areas such as blinding and missing data.

### 3.4. Effectiveness Outcomes

#### 3.4.1. Exercise Capacity

**Figure 4:**
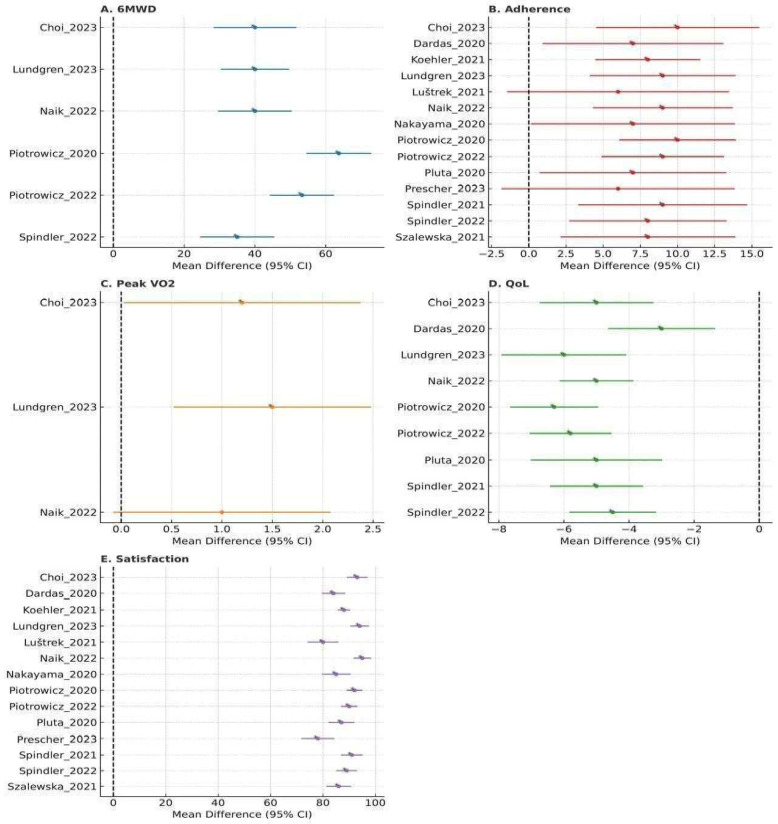
Forest plot for 6-Minute Walk Distance, Peak VO_2_, Quality of Life (QoL), Adherence, Satisfaction mean differences with 95% confidence intervals for the respective studies [19,20,21,22,23,24,25,26,27,28,29,30,31].

The meta-analysis for 6MWD therefore included 6 RCTs (total *n* = 3510) comparing telehealth CR with usual care (Figure 4). No RCT directly compared telehealth CR with centre-based CR for 6MWD, and these comparisons are summarized narratively. Subgroup analysis by intervention duration (≤3 months vs. >3 months) did not show a statistically significant test for subgroup differences. A sensitivity analysis restricted to RCTs with Jadad score ≥ 3 yielded similar pooled SMDs and heterogeneity estimates (Supplementary Table S2), indicating that the effect on 6MWD was robust to trial quality.

#### 3.4.2. Cardiac Function

Five studies assessed LVEF or NYHA class. Choi et al. (2023) [26] reported improved NYHA class (*p* = 0.02) and diastolic function (*p* = 0.024). LVEF improvements were inconsistent.

#### 3.4.3. Clinical Events

Prescher (2023) [28] reported reduced hospitalizations (HR 0.76, *p* = 0.04). No mortality benefits were observed.

### 3.5. Patient-Centered Outcomes: The Findings of Patient Centered Outcomes Were Summarized in Table 3 and Figure 4

The Table 3 summarizes the findings of patient centered outcomes. The pooled analysis demonstrated that telehealth-delivered rehabilitation produced clinically meaningful benefits across multiple functional and patient-reported outcomes. Six studies assessing functional capacity showed a significant improvement in 6-Minute Walk Distance (6MWD), with a small-to-moderate pooled effect (SMD = 0.35, *p* < 0.001), corresponding to an absolute gain of approximately 19–25 m. Although improvements in peak aerobic capacity (peak VO_2_) were modest and did not reach statistical significance (SMD = 0.20, *p* = 0.12), some individual studies reported favorable changes in specific subgroups. Quality of life outcomes were consistently improved across eight studies, indicated by a significant pooled effect (SMD = 0.28, *p* = 0.002) and reflected in better SF-36 and KCCQ scores. Telehealth interventions also demonstrated strong behavioral engagement, with adherence rates ranging from 70–92% and consistently higher than control groups (e.g., 88.7% vs. 80.2%). Patient satisfaction was similarly high, reported in 12 studies, with 75–96% of participants endorsing positive usability, acceptability, and safety. Collectively, these findings support the effectiveness and feasibility of telehealth-based rehabilitation across key clinical, functional, and experiential domains.

**Table 3:** Summary of Outcomes.

Outcome	Studies (*n*)	Telehealth Effect	Key Findings
6MWD	6	SMD 0.35 (*p* < 0.001)	Significant improvement (19–25 m)
Peak VO_2_	3	SMD 0.20 (*p* = 0.12)	Modest improvement in subsets
QoL	8	SMD 0.28 (*p* = 0.002)	Improved SF-36, KCCQ scores
Adherence	12	70–92%	Higher than controls (e.g., 88.7% vs. 80.2%)
Satisfaction	12	75–96%	High usability and safety reported

Note: Each outcome’s study count reflects only the studies reporting that specific outcome, based on 14 RCTs.

#### 3.5.1. Quality of Life

Eight studies reported QoL (SF-36, KCCQ, MLHFQ). Telehealth CR improved QoL (SMD 0.28, 95% CI 0.10–0.46, *p* = 0.002, I^2^ = 50%) compared to usual care. Piotrowicz et al. (2020) [19] reported a 2.7-point SF-36 physical score increases (*p* = 0.008).

Overall, eight RCTs (*n* = 3650) contributed to the QoL meta-analysis, assessing SF-36, KCCQ, or MLHFQ (Figure 4). When stratified by comparator, telehealth CR showed small-to-moderate improvements versus usual care, whereas effects versus centre-based CR were neutral. App-based interventions (4 trials) demonstrated numerically larger QoL gains (SMD~0.40) than telephone-based programs (3 trials; SMD~0.20), but the test for subgroup differences was not statistically significant, likely owing to limited power. Intervention duration (≤3 months vs. >3 months) similarly did not significantly modify the effect. Sensitivity analyses excluding trials with Jadad score < 3 produced almost identical pooled effect estimates and I^2^ values (Supplementary Table S2).

#### 3.5.2. Adherence

Twelve studies reported adherence (70–92%), higher than controls (e.g., Piotrowicz et al., 2020 [19]: 88.7% vs. 80.2%, *p* = 0.002). Prescher (2023) [28] reported 89.1% vs. 82%.

#### 3.5.3. Satisfaction

Twelve studies reported satisfaction (75–96%). Lundgren et al. (2023) [23] noted 96% felt safe, Prescher (2023) [28] 79.7–80%.

### 3.6. Subgroup and Sensitivity Analyses

Pre-specified subgroup analyses were conducted for QoL according to telehealth modality (app-based vs. telephone-based), comparator type (usual care vs. centre-based CR), and intervention duration (≤3 months vs. >3 months). App-based programs tended to produce larger QoL improvements than telephone-based programs, but none of the tests for subgroup differences were statistically significant. Comparator type and intervention duration similarly did not yield statistically significant subgroup effects. Sensitivity analyses excluding RCTs with Jadad score < 3 produced pooled SMDs and heterogeneity estimates very similar to the main analyses, indicating that the overall findings were robust to trial quality (Supplementary Table S2).

## 4. Discussion

This systematic review and meta-analysis shows that telehealth-based cardiac rehabilitation for heart failure offers clinically meaningful benefits over usual care and outcomes comparable to centre-based programs. Across 14 randomized controlled trials, telehealth CR produced small-to-moderate improvements in 6-min walk distance and health-related quality of life, with high adherence and satisfaction. However, effects on peak VO_2_ were inconsistent, and no mortality benefit was demonstrated, highlighting that telehealth CR should be viewed as a complementary strategy rather than a replacement for guideline-directed medical therapy.

Telehealth-based CR significantly outperformed usual care across multiple outcomes and demonstrated equivalence to center-based CR. For exercise capacity, it improved 6MWD (SMD 0.35, 95% CI 0.15–0.55, *p* < 0.001), with Lundgren et al. (2023) and Szalewska et al. (2021) reporting respective gains of 19 m and 25 m [23,25]. Although Piotrowicz et al. (2020) reported a modest but significant increase in peak VO_2_ (0.95 mL/kg/min, *p* < 0.001), the overall meta-analysis did not show a significant effect (SMD 0.20, *p* = 0.12) [19]. Cardiac function outcomes were variable; Choi et al. (2023) [26] reported significant improvements in NYHA functional class (*p* = 0.003) and diastolic function (*p* = 0.024), though LVEF remained unchanged. Similarly, Piotrowicz et al. (2020) found modest gains in peak VO_2_ (+0.95 mL/kg/min, *p* < 0.001) and quality of life (SF-36 physical score +2.7), despite no long-term mortality or hospitalization benefit [19,26]. Adherence to telehealth-based cardiac rehabilitation ranged from 70% to 92%, with Piotrowicz et al. (2020) [19] reporting 88.4% full adherence during a 9-week hybrid telerehabilitation program, significantly higher than in usual care. Similarly, Spindler et al. (2021) observed comparable adherence levels between telerehabilitation (85%) and center-based programs (88%), indicating telehealth CR can match traditional models in patient engagement [19,27]. Patient satisfaction with telerehabilitation was high, ranging from 75% to 96% across studies. In Lundgren et al. (2023), 96% of participants reported feeling safe during supervised, real-time, home-based exercise sessions, and were motivated to continue exercising independently [23]. In the single-arm feasibility study by Leenen et al. (2024) [32] not included in the pooled analyses, the digital health platform used alongside home hospitalization demonstrated good engagement, with 79% usage across hospitalization days. Healthcare professionals reported moderate satisfaction, with 54–46% expressing satisfaction at mid- and end-points, though the platform was not fully integrated into clinical workflows [32].

Improvements in exercise capacity observed in telehealth CR trials (e.g., 19–25 m 6MWD) are consistent with findings from Taylor et al. (2022), who reported mean gains of 20–30 m in home-based cardiac rehabilitation programs [33]. These effects are likely attributable to structured exercise regimens, as seen in Piotrowicz et al. (2020), where hybrid telerehabilitation combined aerobic and resistance training with remote supervision to optimize outcomes [19]. These findings are consistent with the well-established physiological benefits of exercise training in patients with chronic heart failure, including improved peripheral muscle function and hemodynamic responses. Notably, app-based interventions with real-time feedback and interactive features showed greater effects on QoL (SMD 0.40), supporting the role of patient-centered technology in enhancing engagement and self-management (Leslie et al., 2021) [9].

Importantly, the absence of a statistically significant pooled effect on peak VO_2_ and the lack of demonstrated mortality benefit across the included trials indicate that telehealth CR, while beneficial for functional status and patient experience, may not yet translate into improvements in hard clinical endpoints. Several trials were underpowered for mortality and rehospitalization outcomes, and follow-up rarely exceeded 12 months. Real-world implementation may further limit impact due to variability in digital literacy, incomplete integration of telehealth platforms with existing clinical workflows, and uncertainties around reimbursement. These factors highlight the need for pragmatic, longer-term studies that embed telehealth CR into routine care pathways.

Adherence to telehealth-based cardiac rehabilitation (CR) was generally high, ranging from 70% to 92%, consistently surpassing that of usual care and closely matching center-based programs. For instance, Piotrowicz et al. (2020) reported that 88.4% of patients in the hybrid comprehensive telerehabilitation (HCTR) group completed ≥80% of prescribed sessions, demonstrating strong feasibility and patient acceptance of structured, remotely supervised CR interventions [19]. Similarly, Spindler et al. (2022) observed increased digital health engagement, attributing the positive adherence outcomes to supportive technology design and patient motivation [29]. In contrast, Luštrek et al. (2021) found lower adherence (approximately 70%), which was potentially linked to the complexity of the HeartMan system interface and varying levels of digital literacy among users [22]. These findings underscore the importance of intuitive, patient-centered telehealth platforms for optimizing adherence and scalability.

Collectively, telehealth-based CR demonstrates robust clinical utility—consistently outperforming usual care and achieving equivalence with center-based CR across key outcomes. Improvements in functional capacity (6MWD gains of 19–25 m) and quality of life (SMD 0.28) align with results from Piotrowicz et al. (2020) and Spindler et al. (2021), who noted similar or superior performance in hybrid and home-based models [19,27]. High adherence rates, ranging from 70% to 92%, and satisfaction levels up to 96% (e.g., Lundgren et al., 2023) underscore the acceptability and feasibility of remote CR—particularly in patients with logistical barriers or reduced access to traditional programs [23]. Notably, Spindler et al. (2022) and Simon-Leslie et al. (2021) emphasized the motivational value of interactive digital platforms, which promote engagement and long-term behavior change through real-time feedback and personalized education [9,29]. Although Piotrowicz et al. (2020) did not observe long-term reductions in mortality or rehospitalization, early functional gains and scalable implementation support its broader adoption [19]. Taken together, these findings affirm telehealth CR as a viable, effective, and accessible alternative to in-person rehabilitation, especially for high-risk or underserved heart failure populations.

From a health-system perspective, telehealth CR has the potential to reduce travel burden and extend the reach of rehabilitation services, particularly in rural or underserved regions. Although formal cost-effectiveness analyses were not consistently reported in the included trials, prior evaluations of home-based and telehealth CR suggest that remote delivery can be cost-neutral or cost-saving once reductions in travel, facility use, and staff time are accounted for. Future studies should incorporate standardized economic evaluations and implementation outcomes—including scalability, acceptability, and fidelity—to better inform policy and reimbursement decisions.

### 4.1. Limitations and Future Recommendations

This review has several limitations. First, significant heterogeneity existed among the telehealth interventions—ranging from telephone support to advanced mobile applications—which limited the ability to conduct robust meta-analyses. Studies such as Choi et al. (2023) lacked complete demographic data, including age and gender stratification, hindering generalizability, especially to underrepresented populations like women and non-Caucasian groups [26]. Moreover, short follow-up durations (typically 9 weeks to 12 months) constrained insights into long-term adherence, morbidity, and mortality impacts. Additionally, moderate risk of bias was noted in several studies due to absence of blinding or low methodological quality. Notably, the digital literacy and usability of platforms varied; as Thomas et al. (2021) emphasized, poorly designed interfaces or lack of personalization can reduce engagement and limit clinical benefit [34].

Reporting quality also varied; for instance, many studies did not provide device-level technical specifications (e.g., sampling frequency, sensor type, monitoring algorithms), which limits the ability to evaluate the impact of technology fidelity on clinical outcomes. Adherence definitions were inconsistent across RCTs, reducing comparability and undermining pooled estimation of program completion rates. Variations in centre-based CR comparators—differing in duration, exercise intensity, and educational content—may have further influenced comparative effect estimates. Methodological inconsistencies in QoL measurement (SF-36, KCCQ, MLHFQ) and incomplete reporting on whether scale directionality was harmonized across studies also pose interpretational challenges.

Future research should standardize telehealth CR protocols, reporting frameworks (e.g., TIDieR, CERT), and core outcome sets to enhance reproducibility. Large, long-term RCTs (≥12–24 months) are needed to evaluate sustained clinical effects, cost-effectiveness, and implementation outcomes. Greater inclusion of women, older adults, and underserved populations is essential to improve external validity. Future trials should also compare different digital delivery models (fully remote vs. hybrid), evaluate advanced components such as AI-supported monitoring and adaptive exercise algorithms [35], and incorporate strategies to improve digital literacy through user-centred design, caregiver-supported onboarding, and simplified interfaces.

### 4.2. Implications

Clinical Practice: Telehealth CR should be integrated into HF management, particularly for patients with geographical or mobility barriers. Hybrid models, such as HCTR, combining in-person initiation with remote follow-up, can optimize exercise and psychological support. Training healthcare providers in telehealth delivery will ensure program fidelity and patient safety.

Policy: Policymakers should consider reimbursement for telehealth CR, in line with evolving post-COVID-19 models of care. Investments in rural broadband and device subsidies will address access inequities, enabling scalability for underserved populations.

Technology: Developers should focus on intuitive app interfaces and wearable devices with real-time feedback to enhance adherence and engagement, especially for older or low-tech-literacy patients. Standardizing telehealth platforms will facilitate broader adoption.

## 5. Conclusions

Telehealth-based CR appears effective for many patients with HF, improving exercise capacity (6MWD), QoL, adherence, and satisfaction, with outcomes that are generally comparable to centre-based CR and superior to usual care. It addresses important access barriers and offers a potentially scalable, patient-centred alternative to conventional delivery models. Healthcare systems should consider integrating telehealth CR into heart failure management pathways, accompanied by appropriate policy and infrastructure investments. Longer-term trials in diverse and under-represented populations are needed to clarify its impact on hard clinical outcomes and cost-effectiveness.

## Figures and Tables

**Table 1:** Characteristics of Included RCTs Studies only (*n* = 14).

Authors (Year)	Country	Sample Size	Patient Characteristics	Telehealth Intervention	Control	Duration
Piotrowicz et al. (2020) [19]	Poland	850	NYHA I–III, LVEF ≤40%, age 62.4, 88.6% male	9-week HCTR	Usual care	9 weeks
Nakayama et al. (2020) [20]	Japan	236	LVEF < 50%, post-hospitalization	Remote CR with telephone support	Outpatient CR/no CR	30 days
Koehler et al. (2021) [21]	Germany	710	PHQ-9 ≥ 10, CHF	Telemonitoring (ECG, BP, weight)	Usual care	12 months
Luštrek et al. (2021) [22]	International	56	CHF	HeartMan app + wristband	Usual care	Not specified
Lundgren et al. (2023) [23]	Norway	61	NYHA II–III, age 67.6, 18% female	3-month high-intensity telerehabilitation	Usual care	3 months
Pluta et al. (2020) [24]	Poland	850	NYHA I–III, LVEF ≤ 40%, age 62.4, 88.6% male	HCTR with CIED monitoring	Usual care	9 weeks
Szalewska et al. (2021) [25]	Poland	850	NYHA I–III, LVEF ≤ 40%, age 62.4, 88.6% male	9-week HCTR	Usual care	9 weeks
Choi et al. (2023) [26]	South Korea	74	HF	Heart Failure-Smart Life app	Usual care	3 months
Spindler et al. (2021) [27]	Denmark	140	HF	Heart Portal telerehabilitation	Center-based CR	12 months
Prescher (2023) [28]	Germany	1538	Age 70.3, 70% male	RPM (weight, BP, ECG)	Usual care	12 months
Spindler et al. (2022) [29]	Denmark	137	HF	Future Patient Program	Center-based CR	12 months
Piotrowicz et al. (2022) [8]	Poland	850	NYHA I–III, LVEF ≤ 40%, age 62.4, 88.6% male	9-week HCTR	Usual care	9 weeks
Dardas et al. (2020) [30]	USA	Not specified	HF	Centralized decision support	Monitoring	Not specified
Naik et al. (2022) [31]	Germany	1538	LVEF ≤ 45%, age 70.3, 70% male	RPM (vital signs)	Usual care	12 months

**Table 2:** Quality Assessment of Included Studies.

Authors (Year)	PEDro Score	Jadad Score	RoB2 Overall Risk
Piotrowicz et al. (2020) [19]	8	4	Low
Nakayama et al. (2020) [20]	6	3	Low
Koehler et al. (2021) [21]	7	3	Low
Luštrek et al. (2021) [22]	6	2	Some concerns
Lundgren et al. (2023) [23]	7	3	Low
Pluta et al. (2020) [24]	7	4	Low
Szalewska et al. (2021) [25]	7	4	Low
Choi et al. (2023) [26]	6	3	Some concerns
Spindler et al. (2021) [27]	7	3	Low
Prescher (2023) [28]	7	4	Low
Spindler et al. (2022) [29]	7	3	Low
Piotrowicz et al. (2022) [8]	8	4	Low
Dardas et al. (2020) [30]	7	2	Some concerns
Naik et al. (2022) [31]	7	4	Low

## Data Availability

The original contributions presented in this study are included in the article/Supplementary Material. Further inquiries can be directed to the corresponding author.

## Supplementary Materials

The following supporting information can be downloaded at https://www.mdpi.com/article/10.3390/medicina62010025/s1, Table S1. Full Electronic Search Strategies for All Databases; Table S2. Summary of predefined subgroup analyses and sensitivity analyses for exercise capacity (6MWD) and health-related quality of life (QoL).
