# Supplementary material for: Telehealth-Based Cardiac Rehabilitation for Heart Failure: A Systematic Review of Effectiveness, Access, and Patient-Centred Outcome
Source: Medicina (Kaunas). 2025 Dec 23;62(1):25. doi: 10.3390/medicina62010025 (PMC12842665; doi:10.3390/medicina62010025)
Supplement: Supplementary file 1 [file medicina-62-00025-s001.zip › medicina-3967167-supplementary.pdf]

Supplementary Table S1. Full Electronic Search Strategies for All Databases.

PubMed (searched: 10 June 2025)

Search string:

("Heart Failure"[Mesh] OR "heart failure" OR "cardiac failure" OR "congestive heart failure")

AND

("Cardiac Rehabilitation"[Mesh] OR "cardiac rehabilitation" OR "exercise training")

AND

(telehealth OR telemedicine OR telerehabilitation OR "remote monitoring"

OR "mobile health" OR mHealth OR "eHealth")

AND

(randomized controlled trial OR randomized OR randomised OR RCT)

Filters: Humans; Adults; English; 2020–2025.

MEDLINE (Ovid) (searched: 10 June 2025)

Search string:

1. Heart Failure/ OR heart failure.tw. OR cardiac failure.tw.
2. Cardiac Rehabilitation/ OR cardiac rehabilitation.tw. OR exercise training.tw.
3. telehealth.tw. OR telemedicine.tw. OR telerehabilitation.tw.
   OR remote monitoring.tw. OR mobile health.tw. OR mHealth.tw.
4. randomized controlled trial.pt. OR randomized.ab. OR randomised.ab. OR RCT.tw.
5. 1 AND 2 AND 3 AND 4

Limit: English language; 2020–2025

CINAHL (EBSCOhost) (searched: 10 June 2025)

Search string:

(MH "Heart Failure+") OR "heart failure"

AND

(MH "Cardiac Rehabilitation+") OR "cardiac rehabilitation" OR "exercise training"

AND

telehealth OR telemedicine OR telerehabilitation OR "remote monitoring"

OR "mobile health" OR mHealth OR "eHealth"

AND

(randomized controlled trial OR randomized OR randomised OR RCT)

Limiters: English; Peer-reviewed; 2020–2025.

EMBASE (Elsevier) (searched: 10 June 2025)

Search string:

('heart failure'/exp OR 'heart failure' OR 'cardiac failure')

AND

('cardiac rehabilitation'/exp OR 'cardiac rehabilitation' OR 'exercise training')

AND

(telehealth OR telemedicine OR telerehabilitation OR 'remote monitoring'

OR 'mobile health' OR mhealth OR 'digital health')

AND

('randomized controlled trial'/exp OR randomized OR randomised OR RCT)

Limit to: English; Humans; 2020–2025.

Web of Science (Core Collection) (searched: 10 June 2025)

Search string:

TS = ("heart failure" OR "cardiac failure")

AND

TS = ("cardiac rehabilitation" OR "exercise training")

AND

TS = (telehealth OR telemedicine OR telerehabilitation

OR "remote monitoring" OR "mobile health" OR mHealth OR eHealth)

AND

TS = (randomized OR randomised OR "randomized controlled trial" OR RCT)

Refined by: 2020–2025; English.

Supplementary Table S2. Summary of predefined subgroup analyses and sensitivity analyses for exercise capacity (6MWD) and health-related quality of life (QoL).

SMD = Standardized Mean Difference. CI = Confidence Interval.

A. **Subgroup Analyses: Quality of Life (QoL)**

Subgroup Variable	Subgroup Levels	No. of Trials	Total Sample (N)	SMD (95% CI)	I^2 (%)	p (Subgroup Difference)	Interpretation
**Telehealth Modality**	App-based	4	[Insert N]	0.40 (0.18–0.63)	42 %	0.18	Larger numerical effect but not statistically significant
	Telephone-based	3	[Insert N]	0.20 (0.02–0.38)	35 %		
**Comparator Type**	Usual care	6	[Insert N]	0.32 (0.12–0.52)	48 %	0.27	No significant difference between comparator groups
	Center-based CR	2	[Insert N]	0.05 (−0.14–0.23)	0%		
**Intervention Duration**	≤ 3 months	5	[Insert N]	0.26 (0.08–0.45)	40 %	0.33	Duration did not significantly modify effect

	> 3 months	3	[Insert N]	0.31 (0.09–0.54)	52 %		

B. Subgroup Analyses: 6-Minute Walk Distance (6MWD)

Subgroup Variable	Subgroup Levels	No. of Trials	Total Sample (N)	SMD (95% CI)	I² (%)	p (Subgroup Difference)	Interpretation
**Telehealth Modality**	App-based	2	[Insert N]	0.38 (0.10–0.67)	45 %	0.24	Numerical differences only
	Telephone-based	4	[Insert N]	0.32 (0.10–0.54)	38 %		
**Comparator Type**	Usual care	5	[Insert N]	0.35 (0.12–0.57)	40 %	0.41	No difference between comparator groups
	Center-based CR	1	[Insert N]	0.01 (−0.30–0.32)	—		
**Intervention Duration**	≤ 3 months	4	[Insert N]	0.34 (0.11–0.57)	39 %	0.50	Effect not dependent on duration
	> 3 months	2	[Insert N]	0.30 (−0.01–0.62)	42 %		

C. Sensitivity Analyses (Excluding Jadad <3).

Outcome	No. of Trials Excluded	New SMD (95% CI)	I² (%)	Interpretation
**QoL**	3 low-quality trials (Luštrek, Dardas, Scherrenberg*)	0.27 (0.09–0.45)	48%	Nearly identical to primary analysis → robust
**6MWD**	2 low-quality trials	0.33 (0.12–0.54)	38%	Very similar pooled effect, stable results
